# The impact of distribution properties on sampling behavior

**DOI:** 10.3389/fpsyg.2025.1597227

**Published:** 2025-09-30

**Authors:** Thai Quoc Cao, Benjamin Scheibehenne

**Affiliations:** Cognition and Consumer Behavior Lab, Karlsruhe Institute of Technology, Karlsruhe, Germany

**Keywords:** search behavior, decision-making, skewed distributions, rare outcomes, estimation accuracy

## Abstract

**Objective:**

People often have their decisions influenced by rare outcomes, such as buying a lottery and believing they will win, or not buying a product because of a few negative reviews. Previous research has pointed out that this tendency is due to cognitive issues such as flaws in probability weighting. In this study we examine an alternative hypothesis: that people’s search behavior is biased by rare outcomes, and they can adjust the estimation of option value to be closer to the true mean, reflecting cognitive processes to adjust for sampling bias.

**Methods:**

We recruited 180 participants through Prolific to take part in an online shopping task. On each trial, participants saw a histogram with five bins, representing the percentage of one- to five-star ratings of previous customers on a product. They could click on each bin of the histogram to examine an individual review that gave that product the corresponding star; the review was represented using a number from 0–100 called the positivity score. The goal of the participants was to sample the bins so that they could get the closest estimate of the average positivity score as possible, and they were incentivized based on accuracy of estimation. We varied the shape of the histograms within subject and the number of samples they had between subjects to examine how rare outcomes in skewed distributions influenced sampling behavior and whether having more samples would help people adjust their estimation to be closer to the true mean.

**Results:**

Binomial tests confirmed sampling biases toward rare outcomes. Compared with 1% expected under unbiased sampling, participants allocated 11% and 12% of samples to the rarest outcome bin in the negatively and positively skewed conditions, respectively (ps < 0.001). A Bayesian linear mixed-effects analysis examined the effect of skewness and samples on estimation adjustment, defined as the difference between experienced /observed means and participants’ estimates. In the negatively skewed distribution, estimates were on average 7% closer to the true mean compared with the observed means (10-sample ∆ = −0.07, 95% CI [−0.08, −0.06]; 20-sample ∆ = −0.07, 95% CI [−0.08, −0.06]). In the positively skewed condition, estimates also moved closer to the true mean (10-sample ∆ = 0.02, 95% CI [0.01, 0.04]; 20-sample ∆ = 0.03, 95% CI [0.02, 0.04]). Still, participants’ estimates deviated from the true mean by about 9.3% on average, underscoring the persistent influence of sampling bias.

**Conclusion:**

These findings demonstrate how search biases systematically affect distributional judgments and how cognitive processes interact with biased sampling. The results have implications for human–algorithm interactions in areas such as e-commerce, social media, and politically sensitive decision-making contexts.

## Introduction

When making decisions, people often possess prior knowledge of an option’s quality or possible outcomes from past experiences. For instance, e-commerce platforms commonly display distributions of past customer ratings as an indicator of a product’s quality. Likewise, in many countries, lottery providers are legally required to disclose the probability of winning for a given prize tier. These disclosures are expected to support more informed and rational decisions. However, despite widespread awareness that winning a lottery jackpot is statistically improbable, global spending on lottery tickets amounts to approximately $250 billion annually (Kim and Oswald, 2021). Similarly, marketing research suggests that rare but highly negative reviews can disproportionately damage a product’s reputation, even when positive feedback is abundant (Wu, 2013). These examples suggest that rare outcomes may be subjectively overweighted in these contexts.

The influence of distribution features, such as the rareness of outcomes, on perception and judgments has garnered substantial research attention. Prospect Theory, a prominent explanatory framework for decision making under risk, posits that individuals disproportionately overweight low probabilities, a tendency attributed to the curvature of the probability weighting function (Kahneman and Tversky, 1977). However, Prospect Theory’s explanation is largely derived from studies on choice behavior in binary gambles, with limited exploration of how individuals gather and process information—processes that may be shaped by distinct biases (Azzopardi, 2021; Schulz-Hardt et al., 2000). Recent research has increasingly focused on how the characteristics of a distribution influence its perception (Ludvig and Spetch, 2011; Ludvig et al., 2014; Mason et al., 2024; Stewart, 2009). However, further research is required to examine how these characteristics influence sampling behavior, a process that precedes perception (Hills et al., 2010). Without examining sampling behavior, the biases observed in lottery-like gambles—commonly attributed to cognitive factors—may instead reflect residual effects of biased sampling patterns. For example, Niese and Hütter (2022) demonstrated that the negative framing effect, traditionally explained by Prospect Theory through motivational biases such as loss aversion, can also be explained by sampling processes—where negative framing prompts individuals to retrieve more negative information about an option, leading to framing-dependent biases. This underscores the important role of sampling behavior in reexamining previously established psychological phenomena. In other words, sampling biases could shape cognitive processes such as perception (Johnson and Tversky, 1984; Olschewski et al., 2024; Walters et al., 2023), and preferences (Dohmen et al., 2018; Mallpress et al., 2015; Weber, 2010) which then in turn influence decision outcomes. This suggests a potential causal pathway in which sampling behavior influences perception, ultimately leading to decision biases such as overweighting of rare events. By concurrently analyzing both sampling behavior and information perception, this study seeks to disentangle these components with a special focus on overweighting of rare events.

Evidence from multiple studies indicates that search behavior is sensitive to both external and internal influences. For instance, Biella and Hütter (2024) demonstrated that sampling strategies vary with motivational context: when individuals are driven by interest, they tend to truncate sampling after encountering early counter evidence, whereas in disinterested contexts, they sample more extensively and systematically. External factors, such as rare events, also attract disproportionate attention during information search—even when the shape of the distribution is known. For example, research on lottery buyers suggests that their decision to purchase risky lotteries is driven by a preference for skewness rather than risk, leading them to overweight the observations of a few jackpot winners while neglecting the vast majority of losers in a highly positively skewed environment (Åstebro et al., 2015; Garrett and Sobel, 1999). In contrast, in negatively skewed environments like online reviews, consumers may react negatively to skewness, giving disproportionate attention to a few negative reviews while downplaying many positive ones (Jung et al., 2020; Wu, 2013). Despite the seemingly contradiction, both examples hint at an overweighting of rare outcomes in the decision-making process. While these studies did not experimentally test the influence of these distributional properties on sampling behavior, and the cause of the overweighting of rare values remains unclear, they suggest that sampling behavior inevitably distorts perception by amplifying rare outcomes.

Sometimes, humans also ignore the probability of an event regardless of its consequence, a phenomenon termed ‘probability neglect’ by Sunstein (2003). This can lead individuals to downplay dangerous risks, such as a lightning strike in a storm, while overestimating another risk with a similar probability, such as a terrorist attack. The key difference lies in how people gather and process information about these risks.

In the case of terrorism, sensational media coverage or recommendation systems driven by attention economy often increases people’s exposure to such news, making the risk feel disproportionately large. In contrast, while lightning strikes are also rare, they receive much less media coverage than terrorism due to the lack of sensationalism. Consequently, although humans are often biased toward rare events, they may also downplay similar extreme risks when their experience to that risk is limited (Sunstein, 2003). This distinction underscores the importance of understanding the context and sampling behaviors driving different types of biases in risk perception and decision-making.

Here, we aim to investigate how the skewness of a distribution influences individuals’ sampling behaviors and their perception of options. By hypothesizing that individuals pay more attention to rare events, as evidenced by the greater frequency of their sampling, we seek to understand seemingly irrational behaviors, such as excessive lottery spending or exaggerated reactions to rare negative reviews. Prior to presenting our study design, we will review relevant literature on how biased sampling and knowledge of distributional properties influence decision-making.

### Biased sampling behavior toward rare events

Research on human sampling behavior spans multiple domains, though findings often diverge across paradigms (Von Helversen et al., 2018). For instance, in foraging studies, where search incurs energy and opportunity costs, individuals tend to explore locally before moving on to the next food patch (Charnov, 1976; Hills, 2006; Hills et al., 2015). The distinctive feature of this paradigm is the high cost of switching (e.g., energy expenditure to travel *between* food patches), which compels the agent to weigh the trade-offs between continuing with the current depleting option and exploring alternatives with a great cost (Von Helversen et al., 2018). A different paradigm is needed to capture the nature of online information search, where information is abundant, and switching costs between options are minimal. The decision-from-experience (DfE) paradigm offers a more suitable framework, focusing more on how people collect, perceive, and evaluate information from multiple options (Hertwig et al., 2004). In typical DfE experiments, participants sample between two options, each drawing a random outcome from a distribution or pre-generated number sequence, to assess which option is preferable or their willingness to pay (Johnson and Tversky, 1984; Olschewski et al., 2024; Walters et al., 2023). Using the DfE paradigm, Hills et al. (2013) demonstrated that as the number of available options increases, individuals sample a broader range of options but gather fewer samples from each. This sampling pattern appears across various contexts, including consumer psychology (Levav et al., 2012), goal-directed search (Hills et al., 2010; Vul et al., 2014), and social perception (Biella and Hütter, 2024). Studies using eye-tracking in search behavior have shown that individuals spread their attention across numerous alternatives, focusing more closely on options with prominent or favorable features (Bella-Fernández et al., 2022; Rajsic et al., 2015).

One important drawback of the sampling paradigm is that in both, foraging and information search studies, the shape of the distribution is typically unknown. This lack of information complicates efforts to isolate the influence of distributional shapes such as skewness on sampling behavior. By contrast, in contexts such as online reviews or lotteries, individuals often have a general overview of the distribution’s shape while searching for additional information (e.g., a histogram of star ratings). To mimic this scenario, we designed an environment where participants are ﻿shown the shape of different histograms with five bins and the probability of each bin, ﻿but the range of values remained hidden. This setup necessitates information search to learn the objective mean (i.e., the true mean) of the distribution. In such situations, where individuals have access to the distribution’s shape but not its range and aim to estimate an option’s true value, they might ideally employ stratified sampling—an efficient and unbiased strategy that allocates samples based on the probability of each outcome (a more formal description of the context and supporting proof can be found in the Appendix). By contrast, an excessive allocation of samples to rare events, particularly in skewed distributions, may lead to distorted perceptions of the true mean.

﻿To further examine how people prioritize information search, we consider the hypothesis that individuals focus more on rare and extreme outcomes, rather than just rarity alone. In an environment where all outcomes are equally probable, a search strategy driven purely by rarity would result in an even distribution of sampled outcomes. However, if individuals disproportionately sample values at the distribution’s upper and lower bounds, despite their equal likelihood, this would suggest that information search is guided by the extremity of outcomes rather than rarity alone.

Overall, we hypothesize that individuals exhibit biased sampling behavior toward rare outcomes, even when they are aware of the distribution’s shape. This study aims to examine how individuals allocate their search efforts across different outcome categories when provided with explicit knowledge of the distribution.

*H*1: ﻿When facing skewed distributions, participants will over-sample the rarest outcome relative to its actual probability, whereas for uniform distributions, their sampling will resemble the true distribution.﻿﻿﻿

### Perception of biased experienced means

Another important aspect is how people perceive the information they sample. Past research employed estimation tasks to assess how people perceive a sequence of outcomes. Unlike valuation and choice tasks, estimation tasks are incentivized based on accuracy, thus not influenced by risk preferences (Olschewski et al., 2021, 2024). Studies have found that people often underestimate the mean of a presented number sequence, possibly due to a “compressed mental number line”—a cognitive bias that leads to estimates lower than the actual mean of the distribution (Oberholzer et al., 2021). However, it is important to note that in these tasks, the number sequences were presented to participants as a continuous stream on the screen rather than requiring an active sampling process, thereby removing the influence of any sampling biases. Consequently, it remains unclear how sampling biases affect the perception of a distribution’s true mean.

When individuals draw information from a distribution, the limited number of observations they encounter and how they allocate their search may lead to two distinct concepts of the mean. The first is the *true mean*, representing the central tendency of the underlying distribution. The second is the *experienced mean*, derived by averaging the subset of observations they have encountered. In skewed distributions, biased sampling behavior is expected to produce a biased experienced mean. For instance, oversampling rare outcomes in a negatively skewed distribution may result in an experienced mean lower than the true mean, whereas oversampling rare outcomes in a positively skewed distribution may lead to an experienced mean higher than the true mean. To the extends that individuals infer the true mean based on their experienced mean, any bias in the experienced mean will systematically lead to over- or underestimation of the true mean. Therefore, we hypothesize that:

*H*2a: For negatively skewed distributions, the experienced mean will be smaller than the true mean (negative bias).

*H*2b: For positively skewed distributions, the experienced mean will be larger than the true mean (positive bias).

*H*2c: For uniform distributions, there will be no systematic difference between the experienced mean and the true mean (no bias).

While the experienced mean depends on sampling behavior, how people perceive and adjust for it is influenced by cognitive factors. Individuals adapt their valuations not only based on external factors such as social norms (Fleischhut et al., 2022) but also on internal factors, including confidence (Olschewski and Scheibehenne, 2024; Soll et al., 2019; Yeung and Summerfield, 2012). These types of adjustments require a basic level of causal reasoning about the environment or metacognitive awareness of the decision-maker’s own limitations (Olschewski and Scheibehenne, 2024). For example, consider a real-world scenario in which ﻿someone asks a friend to estimate the price of an item they purchased during a Black Friday sale. If the friend underestimates the typical price of the item, their reasoning may involve internal adjustments—such as accounting for the likelihood of discounts during sales events. Crucially, this type of adjustment relies on prior knowledge or familiarity with Black Friday, suggesting that better prior information facilitates more accurate adjustments.

By extension, we hypothesize that people will recognize and adjust for sampling biases when sampling from skewed conditions, shifting their estimates closer to the true mean, particularly as the sample size increases. This hypothesis is grounded in research on metacognition adjustment (Olschewski and Scheibehenne, 2024; Yeung and Summerfield, 2012), and rational learning (Le Mens and Denrell, 2011; Olschewski et al., 2024) which suggests that individuals adjust their judgment based on internal factors such as their confidence, and the awareness of their cognitive and behavioral limitations. While participants may over-sample from rare outcomes, we expect that mental adjustments away from the experienced mean will improve estimation accuracy when people have more experience with the skewed distributions due to observing more samples. We do not expect an increase in estimation accuracy with sample size in the uniform condition because here we do not expect the experienced mean to deviate from the true mean in the first place.

*H*3: Increasing the number of samples from skewed distributions will reduce the difference between participants’ estimates and the true mean. However, this effect will not be observed in the uniform condition.

## Method

### Study design

Participants took part in an online shopping simulation designed to examine how they sample information to estimate the average score of customer reviews. The task consisted of multiple trials. In each trial, participants were presented with a histogram of customer ratings ranging from 1 to 5 stars, where 5 represented the highest rating. They were informed that each star rating was associated with a text “review” that was converted into a numerical positivity score ranging from 0 (very negative) to 100 (very positive). The cover story was designed to make the instructions more intuitive compared to an abstract task involving statistical distributions and histograms. We also explicitly informed participants that both the ratings and positivity scores were entirely computer-generated for the experiment and not derived from real customer reviews to eliminate potential inference bias.

There were three within-subject conditions, each consisting of different histogram shapes: negatively skewed, positively skewed, and uniform (four trials per condition). The uniform condition served as a non-skewed baseline to determine whether participants focused just on rare events or a combination of rare *and* extreme events (Mason et al., 2024). The skewed conditions aimed to assess how distribution shape ﻿influenced sampling behavior and experienced means (H1 and H2).

During each trial, participants could click on individual bins of the histogram to sample a positivity score corresponding to that star rating (e.g., clicking on the “1-star” bin would reveal the positivity score of a simulated customer who rated the product with 1 star). However, they had a limited number of samples they could collect during each trial.

To assess the impact of number of samples, participants were randomly assigned to one of two between-subject conditions: one group could collect 10 samples, while the other group could collect 20 samples from the distribution. This manipulation was designed to investigate whether participants adjusted their sampling strategies and estimations when given more samples (H3).

Participants’ primary task was to estimate the product’s average positivity score based on their samples. To incentivize accuracy, participants were offered a potential reward of up to £8, depending on how closely their final estimate matched the true average positivity score.

### Stimuli creation

The experimental design and hypothesis were preregistered on AsPredicted.^1^ To create the stimuli, we used three distinct beta distributions, each representing a specific shape condition: negatively skewed *β*(3, 1), positively skewed *β*(1, 3), and uniform *β*(1, 1). These distributions were then scaled by a random multiplier between 50 and 100 for each trial to mask the true range of positivity scores and to prevent participants from inferring the underlying distribution’s exact range based on previous trials.

**Figure 1:**
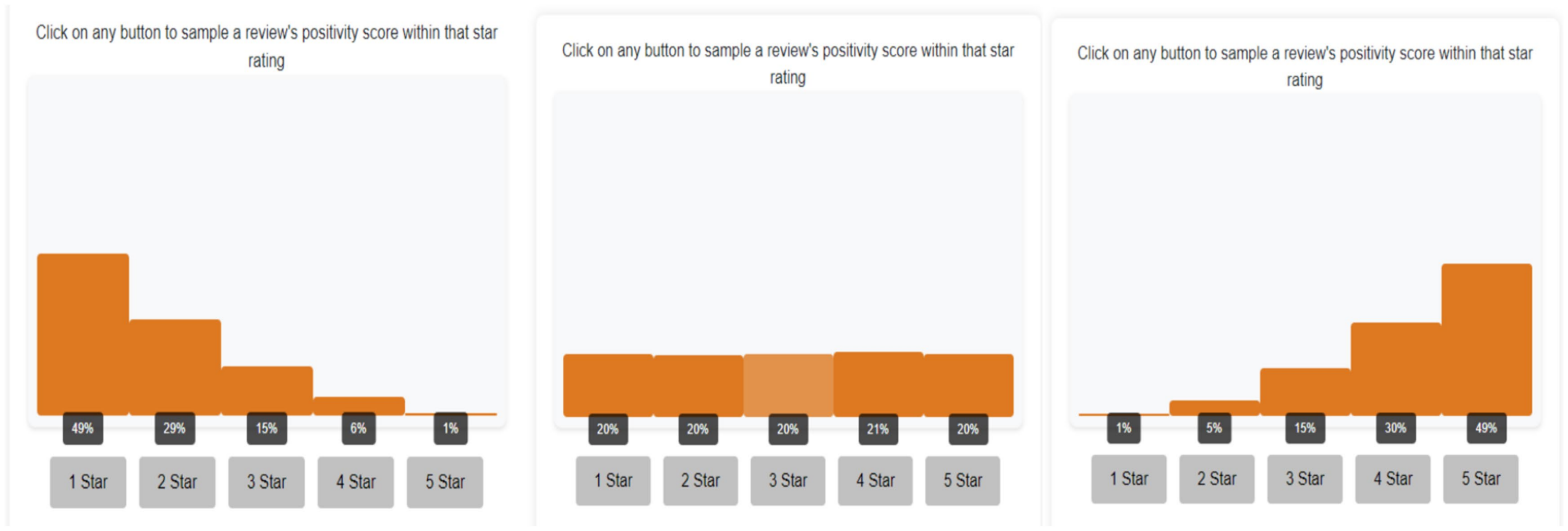
Screenshot of different distributional shape conditions. From left to right: positively skewed condition, uniform condition, and negatively skewed condition. Clicking on each button below draws a sample from the respective interval of the underlying distribution.

Histograms presented to participants were created by binning the beta distributions into five intervals, with each bin displaying the probability of receiving a sample from that interval if drawn randomly (see Figure 1). When participants sampled from a bin, a score was drawn from the corresponding interval in the continuous beta distribution and shown below the histogram.

### Procedure and incentives

Following the pre-registered sample size, we recruited 180 participants through Prolific (2024) to take part in our online experiment. The task was implemented using Otree (Chen et al., 2016). Participants were randomly assigned to either the 10- or 20-sample condition. At the experiment’s start, participants were briefed on sampling rules and positivity scores and informed of a potential £8 bonus based on the accuracy of their estimates in one randomly selected trial.

Participants began with a practice trial featuring a randomly selected distribution shape to familiarize themselves with the task. After sampling from the histogram, they provided an estimate for the average positivity score and proceeded to the next round. Following the practice trial, participants completed comprehension checks to ensure they understood the task before beginning the main task. ﻿In the main task, they completed three within-subject conditions presented in randomized block order, with four trials per condition. Upon completing all trials, participants provided demographic information. Following the preregistered exclusion criteria, we excluded trials in which only one bin was sampled. We also excluded participants who provided the same estimation in all trials, as well as any participants with two excluded rounds. This resulted in a final sample of 145 participants (Mean_age_ = 39, SD = 12; 47% male, 53% female, 50% in the 10-samples condition).

## Results

### Sampling bias

Binomial tests were conducted to evaluate the hypotheses regarding participants’ sampling biases toward rare outcomes in skewed distributions. In particular, we compared the observed sampling behavior with a simple heuristic of stratified sampling, which is a simple, efficient and unbiased sampling rule in this task (proof in Appendix). In the negatively skewed condition, participants spent 11% of their total sample on bin 1, substantially more than the expected 1% (*p* < 0.001, *95% CI* [0.10, 0.11]). Similarly, in the positively skewed condition, participants sampled from bin 5 (12%) more than suggested 1% by the stratified-sampling heuristic (*p* < 0.001, *95% CI* [0.11, 0.13]). These results provide evidence of a sampling bias toward rare events in skewed distributions. Across both the 10- and 20-sample conditions, participants allocated approximately 10% of their samples to rare events, suggesting a strong and consistent tendency to focus more on these outcomes as compared to stratified sampling.

**Figure 2:**
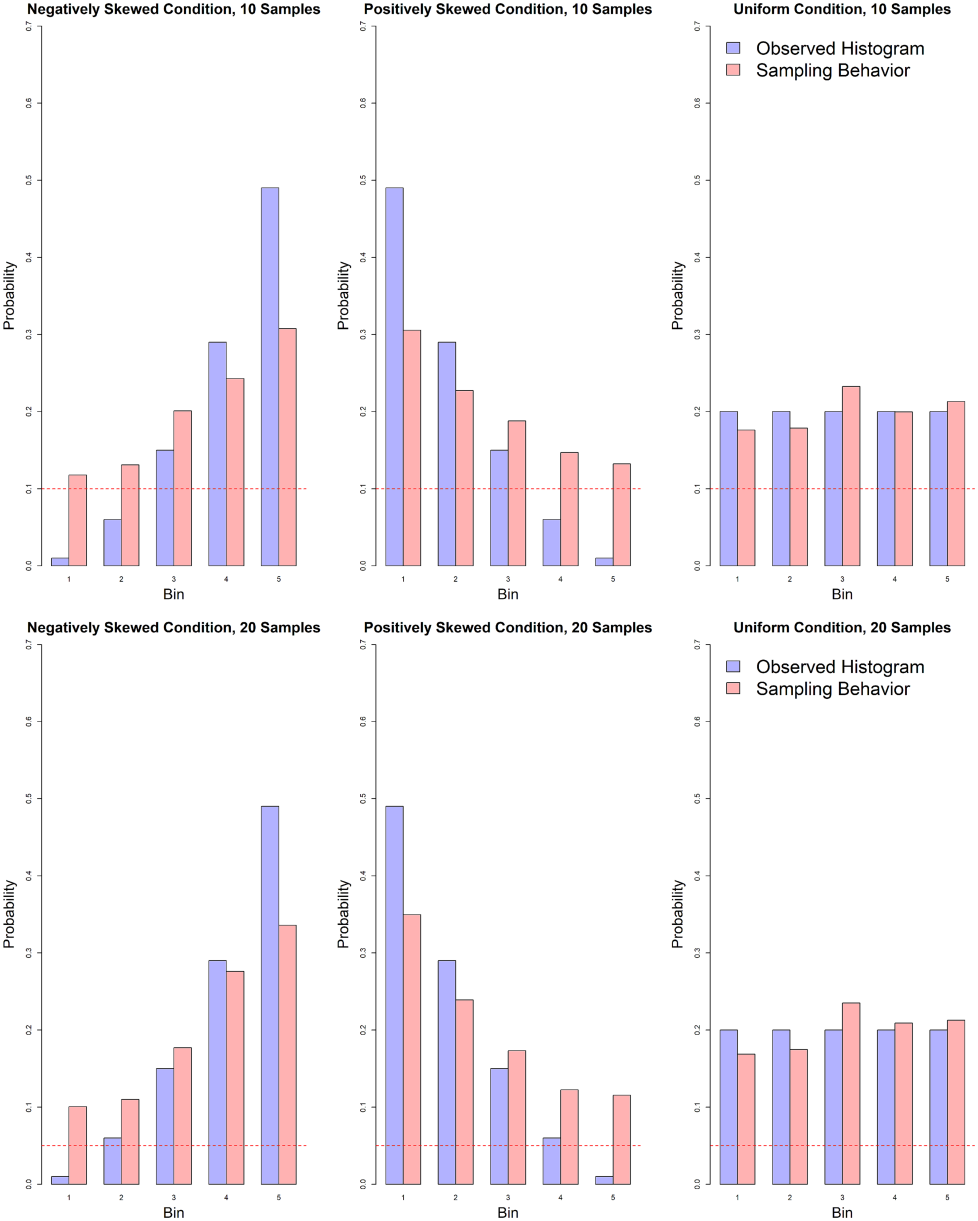
Comparison of the observed histogram (light purple) with participants’ actual sampling behavior (light red) in the 10- and 20-sample conditions. The red dotted line represents the proportion of samples allocated if each option were sampled exactly once.

In the uniform distribution condition, we observed a slight undersampling in bin 1 (*P_bin1_* = 0.17, *95% CI* [0.16, 0.18], *p* < 0.001), and no statistically significant difference in bin 5 (*95% CI* [0.20, 0.22], *p* = 0.063), providing evidence against a strong sampling bias toward a combination of rare *and* extreme events in this condition. However, we observed a small positive bias in bin 3 of the uniform condition (*P_bin3_* = 0.23, *95% CI* [0.23, 0.24], *p* < 0.001). Although this was not predicted in our hypothesis, the oversampling of bin 3 suggests that participants may have employed a simple rule of thumb: sampling from the middle bin to approximate the mean when the distribution shape was uniform. This demonstrate that participants understand the task quite well and adapt their sampling strategies to solve the task (Figure 2).

### Experienced mean and estimation bias

An important aspect of decision-making lies in how individuals perceive and internalize the information obtained through their sampling behavior, particularly in relation to systematic biases across different distributional shapes and levels of search resources. In this section, we focus on our two key hypotheses: the deviation between the experienced mean and the true mean (H2), and the deviations between participants’ estimation and the experienced and true means (H3). To investigate these hypotheses, we constructed a series of Bayesian linear mixed-effects models with a common structure—using the same set of predictors but varying in dependent variables. Models were implemented in R (R Core Team, 2021), using the brms package (Bürkner, 2021). Each model included random intercepts for participants and fixed slopes for the main effects, and was estimated using the default priors for the Gaussian family in brms.

**Figure 3:**
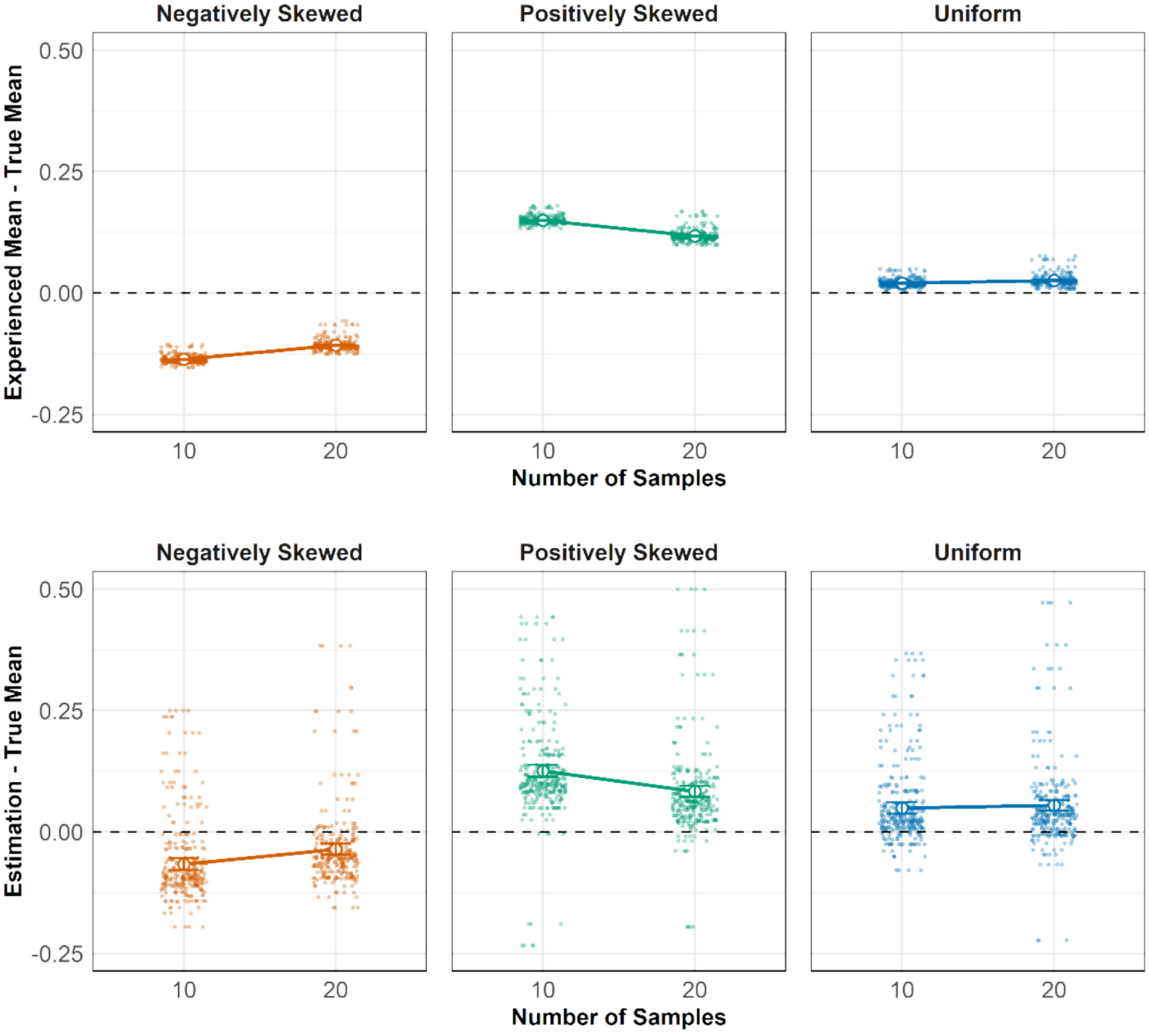
Upper: The effect of distribution shapes and sample sizes on the difference between the experienced mean and the true mean. Lower: The effect of distribution shapes and sample sizes on the difference between participants’ estimates and the true mean. The y-axis in both plots is standardized to the same scale, ranging from −1 to 1. Error bars represent 95% credible intervals.

For H2, a Bayesian linear mixed-effects model was used with sampling bias, defined as the deviation of the experienced mean from the true mean of the distribution, as the dependent variable. The predictors included skewness conditions (negatively skewed, positively skewed, and uniform), sample sizes (10 and 20), and their interaction effect. We first report the experienced mean for the 10-sample condition. In the negatively skewed condition, the sampled mean was below the true mean, yielding a negative sampling bias (*M =* −0.14, *95% CI*: [−0.15, −0.12]). Conversely, in the positively skewed condition, the sampling bias was positive (*M =* 0.149, *95% CI*: [0.148, 0.150]). The model indicated a small effect of sample size, with larger samples (20 vs. 10) reducing sampling bias in both the negatively and positively skewed conditions. The upper panel in Figure 3 shows that the experienced means in both the 10- and 20-sample conditions aligned with our predictions in H2a and H2b: the experienced mean was greater than the true mean in the positively skewed condition (slope above the 0 line) and smaller than the true mean in the negatively skewed condition (slope below the 0 line).

The substantial sample bias in the two skewed conditions suggests that participants would incur a potential incentive loss of 13.5% (£1.08) if they base their estimation solely on the experienced mean. In the uniform condition, positive biases in experienced means were observed in both the 10-sample (*M =* 0.02, *95% CI*: [0.019, 0.021]) and 20-sample conditions (*M =* 0.026, *95% CI*: [0.024, 0.027]). This bias in the experienced mean in the uniform condition highlights the importance of sampling behavior, suggesting that even subtle biases in search behavior could lead to a biased experienced mean.

In the previous analyses, we examined how biased sampling behavior led to deviations between the experienced mean and the true mean. To test H3, we now compare participants’ estimates with both the experienced mean and the true mean. The first analysis focuses on the deviation of estimates from the true mean across different skew conditions (negatively skewed, positively skewed, uniform), sample sizes (10 and 20), and their interaction effect. In the negatively skewed condition, the deviance of participants’ estimates to the true mean was smaller in the 20-sample condition (*M =* −0.036) than in the 10-sample condition (*M =* −0.067, *95% CI* for difference: [−0.058, −0.004]). Likewise, the positively skewed 10-sample condition (*M =* 0.126) showed a larger bias than the 20-sample condition (*M =* 0.083, *95% CI* for difference: [0.014, 0.082]). However, in the uniform condition, differences between the 10-sample (*M =* 0.049) and 20-sample (*M =* 0.055) conditions were not different (*95% CI* for difference: [−0.021, 0.007]). These findings support our hypothesis that estimation accuracy improved more in the skewed conditions than in the uniform condition when participants had more samples, although the effect size was modest—an additional 10 samples led to only a 3.6% improvement in accuracy. A linear projection suggests that participants would require approximately 30 samples in the negatively skewed condition and 40 samples in total to achieve an estimate within 5% of the true mean.

Analyzing the difference between experienced means and participants’ estimations—referred to as estimation adjustment—revealed that, on average, participants’ estimates were 7% closer to the true mean compared to their experienced means in the negatively skewed distribution. (10-sample: *∆ =* −0.07, *95% CI* [−0.08, −0.06]; 20-sample: *∆ =* −0.07, *95% CI* [−0.08, −0.06]). In the positively skewed condition, estimates were also closer to the true mean, though to a lesser extent (10-sample: *∆ =* 0.02, *95% CI* [0.01, 0.04]; 20-sample: *∆ =* 0.03, *95% CI* [0.02, 0.04]). Although we observed sampling bias in all three distribution conditions, estimation adaptation only occurred in the skewed conditions but not the uniform condition. A potential explanation is that participants were aware of substantial sampling biases in the two skewed conditions but not the relatively small bias in the uniform condition. These results dovetail with our hypothesis regarding how people adjust their estimates based on the experienced mean. In the skewed conditions, these adjustments led to an average increase of 4.8% in accuracy compared to relying solely on the experienced mean. However, they did not fully eliminate the impact of sampling bias. Despite attempts to adjust, participants’ estimates still deviated from the true mean by an average of 9.3% in the skewed conditions, highlighting the negative consequence of sampling bias in this context.

### A potential explanation for bias sampling behavior

﻿Research on search behavior in online and consumer contexts suggests that exploration increases with lower search costs (Hills and Hertwig, 2010; Hills et al., 2013; Levav et al., 2012). ﻿However, participants in previous experiments often searched under uncertainty with little a-priori information about the outcome distribution. In our experiment, we observed that participants prioritized sampling all five available outcomes, even though they knew that information in the smallest bin of a skewed distribution contributed little to estimating the true mean. While this sampling pattern introduced a bias in the experienced mean, participants partly adjusted for it when estimating the mean, suggesting that they were aware of their sampling bias. This implies that participants’ behavior was not merely an artifact of irrational sampling but rather a strategic approach to information gathering. Previous research have proposed multiple accounts of motivated search, such as attention-induced search (Zilker and Pachur, 2022; Pachur et al., 2018) and confirmation bias in search (Jonas et al., 2001; Suzuki and Yamamoto, 2021). For example, people may focus their search on the most visually salient item, such as one with the brightest color, simply because it captures attention. However, these accounts do not fully explain our findings, as it is unclear for why participants devoted more attention, or exhibited confirmation bias, toward sampling the bin with the lowest probability while comparatively neglecting the bin with the highest probability.

A potential explanation for why participants oversampled the rarest bin is that they prioritized knowing the full range of the distribution to estimate the mean. According to a framework called cognitive fencing (Liu and Scheibehenne, 2025), individuals are more certain that all values within an experienced range are possible, while they remained uncertain about the probability of values outside the experienced range. To reduce this uncertainty, they preferred to allocate samples to discovering the full range rather than focusing solely on accuracy and ignoring the smallest bin, leading to a sampling bias in skewed distributions. An analysis of unique buttons sampled at the participant level showed that out of 145 participants, only three did not sample all five buttons in any of their trials across the three environmental conditions, suggesting that this may have been part of their information search strategy. We utilized the cognitive fencing framework, which accounts for both the shape of the objective distribution that participants observed and the cognitive preference for sampling the entire values range, to model the sampling process of participants. The model attributed participants’ sampling behavior, represented as a histogram f(x) , as a function of g(x) , the presented star rating histogram, and u(x) , a uniform shaped histogram that captures the inherent tendency to sample the full range:


f(x)=(1−w)∗g(x)+w∗u(x)

The parameter w determines the relative weight of the two histograms, quantifying how much the uniform distribution contributes to the final sampling behavior. We set w with a prior of $N(\mu_{w},\sigma_{w}$ ) allowing each participant to have their own parameter. We fitted the model in R using the rstan package (Stan Development Team, 2025) with the No-U-Turn Sampler (NUTS) to estimate 𝑤. Sampling was performed with four chains, each running for 2000 iterations, including 1,000 warm-up iterations. The estimated weight parameter w had a posterior mean of $\mu_{w}$ = 0.51 (95% *CI* [0.21, 0.74]), indicating that the observed sampling behavior was driven by a fair contribution between u(x) and g(x) . This combination means that even though participants were aware of the shape of the distribution, their actual sampling distribution was influenced by both the uniform and the objective histogram equally. Individual differences in this strategy were captured by an estimated $\sigma_{w}$ = 0.22 (95% *CI* [0.21, 0.22]), suggesting substantial variability in how participants weighted the uniform distribution relative to the objective histogram.

Using the parameter w , we can determine whether the observed estimation adjustments stemmed from a response to different distributional shapes (such as an unfavorable/favorable preference for skewness) or from participants’ own strategic adjustments. If participants were aware of their strategy and incorporated it into their estimations, then differences observed across the three distribution conditions would primarily reflect the underlying strategy w . In this case, including w in the model should eliminate the effect of the skewed conditions on estimation adjustment. Conversely, if the distributional shapes independently influenced estimation, its effect should persist even after accounting for w .

To test this, we fitted a Bayesian hierarchical model using absolute sampling adjustment as the outcome variable, with w , distributional shapes, and their interaction as predictors, and participant as a random effect. We specified random intercepts at participants’ level and fixed slopes structure for the main effects, and used the default Gaussian family priors in the brms package. We found only a main effect of w (*b* = 0.15, *SE* = 0.02, 95% *CI* [0.10, 0.19]), with no effects of the shapes of distribution (95% CI [−0.02, 0.02]) or their interaction effect (95% *CI* [−0.05, 0.02]). This supports the idea that while the shape of the distribution influences sampling behavior, its effect on estimation adjustment disappeared when w was taken in account. The extent of adjustment varied according to the magnitude of w , reflecting the premium of sampling the entire range of values, or that participants prioritize sampling all the outcomes, even when it is suboptimal. These results support the hypothesis that participants apply a strategy incorporating sampling all outcomes to have more certainty about the range of values. The estimated influence of w suggests a consistent bias towards an even allocation of samples across bins, particularly when the true distribution is highly skewed.

## Conclusion and discussion

This study examined how distribution shape and sample size impact sampling bias and estimation. We observed significant sampling biases in skewed distributions, with participants disproportionately sampling rare outcomes—bin 1 in the negatively skewed condition and bin 5 in the positively skewed condition. This behavior was consistent across both the 10-sample and 20-sample conditions, leading to biased experienced means due to extra samples of rare outcomes. In addition, we also found a small deviation from the expected sampling behavior in the uniform distribution condition, which contributed to slight deviations in experienced means in this condition as well. Together, these results highlight the importance of sampling behavior in unveiling the number sequence that participants observed.

In our experiment, increasing the number of samples that could be drawn reduced both sampling- and estimation errors in skewed distributions, with participants adjusting their estimates closer to the true mean. This indicates that a larger sample size provides a more accurate representation of the underlying distribution by mitigating the effects of sampling biases. By examining the true mean, experienced mean, and participants’ estimates simultaneously, our study reveals two critical insights regarding the underestimation and overestimation of means in skewed distributions. First, when comparing the true mean with participants’ estimates, we found evidence for overweighting rare outcomes, where people overestimate the mean of positively skewed distributions and underestimate the mean of negatively skewed ones. This pattern aligns with prior research on the perception of skewed distributions (Åstebro et al., 2015; Garrett and Sobel, 1999; Olschewski et al., 2024). The overweighting of rare outcomes in our experiment could be directly linked to participants’ sampling bias, which overrepresents such outcomes—a finding consistent with previous research on decisions from experience (Hills et al., 2013).

When focusing solely on the experienced sequence of numbers sampled by participants and comparing these with their estimates, our results could be interpreted as if they underweighted rare outcomes. Specifically, participants underestimated the experienced mean in positively skewed conditions and overestimated it in negatively skewed conditions because they adjusted their mean estimates. A possible explanation for this pattern of results is that participants’ were aware of their own sampling biases (Olschewski and Scheibehenne, ﻿2024; Soll et al., 2019). This hypothesis is further supported by the fact that in the uniform distribution condition, where sampling bias was minimal, participants’ estimates and their experienced mean was more closely aligned.

Overall, our findings contribute to a better understanding of the overweighting and underweighting of rare events by highlighting how conclusions depend on different points of comparison. Furthermore, our results add to the expanding literature on the joint role of behavioral and cognitive factors in shaping human judgment and decision-making. We observed that sampling behavior is sensitive to features of the choice environment—particularly the presence of rare events—which in turn shapes perception. This aligns with earlier work suggesting that how people sample information can bias what they ultimately perceive and decide (Hertwig and Pleskac, 2010). ﻿Importantly, our results challenge the assumption that people simply rely on what they observe. Instead, we find evidence of cognitive adjustment: individuals appear to recognize the limitations in their own sampling behavior and attempt to correct for them, even if their adjustments are only partially successful.

Our findings resonate with studies that examine the interplay between cognition and sampling behavior in judgment and decision-making. While we did not directly explore the role of higher cognitive function such as motivation, other research has shown that motivational factors can influence how people sample information. For example, Biella and Hütter (2024) found that interest-driven and disinterest-driven search strategies lead to asymmetric sampling: individuals tend to search longer when disinterested and terminate search early when they encounter counterevidence, resulting in more objective information gathering. In our study, sampling bias had a stronger net influence on judgment than cognitive adjustment. ﻿However, Le Mens and Denrell (2011) went even further by demonstrating that even rational sampling processes can yield systematic judgment errors, particularly when individuals prioritize alternatives with more interesting or preferable outcomes. This underscores the critical role of cognitive filters, especially in contexts where individuals have personal stakes or strong prior expectations.

It is important to note that our findings do not imply that people’s search behavior is irrational or inherently biased. In many naturalistic contexts, research has shown that simple heuristics—though sometimes labeled as irrational—can in fact be highly adaptive (Hertwig et al., 2019), often yielding near-optimal solutions (Vul et al., 2014; Lieder and Griffiths, 2019) when search costs and long-term success are considered. In our task, participants performed well under uniform conditions, and we expect similar outcomes in other symmetrical distributions. In the absence of skewness, covering the full range of the distribution, as suggested by the cognitive fencing framework, is a sound strategy for estimating the mean and far easier for humans to implement than random sampling. Moreover, fully exploring the distribution at least once may help rule out alternative hypotheses (e.g., that some surprising outcome is hidden in the data-generating process), thereby reducing effort if the task is encountered again later.

While our results demonstrate a clear bias in sampling behavior under skewed distributions, it is important to emphasize that symmetrical distributions (e.g., Gaussian) are common in nature (Frank, 2009) and may have shaped the heuristics participants employed in our task. From this perspective, the strategy we observed may still enable people to perform well with relatively little effort in many real-world environments. However, this advantage may diminish as skewed distributions become increasingly prevalent and consequential, such as in the distribution of wealth, the occurrence of natural disasters, or information environments shaped by recommendation algorithms and biased organization. This raises important concerns about the potential impact of sampling bias and highlights the need for future research on whether, and how, people adapt their search strategies to such changing environments.

### Future research and implications

Our findings highlight the crucial role of sampling biases in estimation processes, particularly in skewed distributions, and call for further research into how individuals adapt to such biases, especially when they are less overt. Prior research has examined cognitive factors in decision-making (Olschewski et al., 2024), such as memory (Haines et al., ﻿2023; Sahakian et al., 2023), attention (Pleskac et al., ﻿2023), and the integration of numerical information (Oberholzer et al., 2021; Prat-Carrabin and Woodford, 2022). However, our findings underscore the importance of behavioral factors and their critical role in shaping decision outcomes (Bella-Fernández et al., 2022; Mehlhorn et al., 2015; Von Helversen et al., 2018). Behavioral influences, such as sampling biases, have a direct and significant impact on subsequent estimation processes. This provides another perspective on current decision-making theories, which often focus on how information is processed in the brain but treats the informational input as given. The work at hand indicates that cognitive biases can arise at an earlier information sampling stage already. Based on the cognitive fencing framework (Liu and Scheibehenne, 2025), the tendency to oversample the rarest bin in a skewed distribution may have served as a strategy to reduce uncertainty by exploring the full range of values. The model’s parameter helps to explain the observed differences in estimation adjustments across various distribution shapes, emphasizing the interplay between cognitive processes and information search behavior in this task. Additionally, sampling bias alone could not account for the stronger estimation adjustment observed in the negatively skewed condition compared to the positively skewed condition. This result suggests that further investigation into how people perceive and integrate information such as numerical perception (Oberholzer et al., 2021; Olschewski et al., 2024), and information integration strategies (Leuker et al., 2019; Pleskac et al., ﻿2023; Yeung and Summerfield, 2012; Zilker, 2022) may provide valuable insights.

Our findings also have practical implications. For example, in e-commerce, sampling biases, combined with review aggregation algorithms, can distort product perceptions. Consumers often give disproportionate weight to a few negative reviews, which may not accurately represent the broader population, leading to skewed purchasing decisions and potential dissatisfaction (Qahri-Saremi and Montazemi, 2023; Wu, 2013). Similarly, in political voting contexts, the oversampling of rare and extreme opinions can reinforce pre-existing biases, distort perceptions of political candidates, and even sway election outcomes. On social media, where rare events are more likely to go viral, these events can disproportionately shape users’ perceptions of reality, magnify the consequences of fake news or contribute to the spread of false or biased narratives. Although individuals can adjust their opinions and estimations when aware of sampling bias, it remains unclear whether people are aware and can adjust to the bias feedback loop in adaptive systems, recommendation engines, and social media platforms. Without such awareness or intervention, users may remain unaware of the biases shaping their perceptions, resulting in continued misjudgments.

In the context of online information, large language model (LLM) chatbots and AI technologies may be used for reducing biases, but they also hold potential to amplifying bias and misperception. On one hand, LLMs can promote more balanced and objective information search by presenting answers in a comprehensive and impartial manner—even when the user’s initial query or search behavior is biased. In this sense, they can serve as a valuable partner or guide, encouraging users to adopt less biased sampling strategies. LLMs benefit from the law of large numbers: by learning from vast and diverse datasets, their knowledge base far exceeds that of any individual, which can contribute to more balanced responses. Yet, they still inherit and reflect biases present in the training data, sometimes resulting in harmful or morally and practically misleading suggestions (Hanna et al., 2025; Salatino et al., 2025).

While designers often turn to automation to enhance system efficiency and safety, it is important to note that human judgment often becomes even more critical as automation grows in power and ubiquity (Lee and Seppelt, 2023). Therefore, designers of adaptive systems—such as recommendation algorithms and ﻿LLMs—should account for users’ existing sampling biases in order to support more informed decision-making and help mitigate the influence of biased information. Small interventions, such as providing prompts about the prevalence of certain public opinions online or the representativeness of the information collected so far, or using LLMs to detect LLMs-generated contents could potentially help reduce sampling bias of users or bias caused by the system. For example, these prompts could highlight that extreme opinions are not representative of the general public, or the contents being sampled are generated by other LLMs. Additionally, increasing the level of estimation adjustment during the decision-making process may further mitigate these biases.

### Limitations

One limitation is the potential influence of participants’ prior experiences, particularly with positively skewed e-commerce ratings, which may have shaped their sampling and estimation behaviors. For example, frequent exposure to negatively skewed product ratings in e-commerce environments could lead participants to internalize specific real-life problems, such as rating inflations (Aziz et al., 2023; Skreta and Veldkamp, 2009), thereby influencing their judgment in our experimental settings. We selected the context of online shopping because it is familiar to most participants, substantially enhancing task comprehension and reducing rejection rates due to failed comprehension checks.

Additionally, the study focused on two specific sample sizes (10 and 20), which constrains the generalizability of the findings. Increasing the sample size or encouraging more search could potentially reduce sampling biases, but this assumes participants can maintain the same level of attention over a much longer task—an assumption that may not always hold. Because we asked participants to use all available samples before advancing to the next trial, unwanted behaviors such as repeatedly sampling one option to quickly move on to the next trial or randomly clicking might occur. This likely introduced unwanted noise and bias into the data and diminished the reliability of the findings.

Future research could investigate how prior knowledge, such as familiarity with specific rating distributions (e.g., positively skewed ratings common in e-commerce), affects sampling biases and estimation accuracy. Moreover, exploring how learning and experience shape these biases over time could offer valuable insights into the dynamics of human judgment and decision-making. For example, examining how participants adjust their sampling behaviors after receiving feedback on their biases or experiencing varied contexts could help identify effective strategies for mitigating bias across diverse domains, from social media and e-commerce to political decision-making and beyond.

## Data Availability

The raw data supporting the conclusions of this article will be made available by the authors, without undue reservation.

## Footnotes

^1^ https://aspredicted.org/pjdh-74sc.pdf
